# Association of PLGA Microspheres to Carrier Pellets by Fluid Bed Coating: A Novel Approach towards Improving the Flowability of Microparticles

**DOI:** 10.1155/2018/3874348

**Published:** 2018-07-02

**Authors:** André O'Reilly Beringhs, Aline Benedita dos Santos Fonseca, Angela Machado De Campos, Diva Sonaglio

**Affiliations:** ^1^Department of Pharmaceutical Sciences, School of Pharmacy, University of Connecticut (UConn), 69 N Eagleville Rd, Unit 3092, Storrs, CT, USA; ^2^Department of Pharmaceutical Sciences, Center of Health Sciences, Federal University of Santa Catarina (UFSC), Florianópolis, SC, Brazil

## Abstract

Micro- and nanoparticles have been vastly studied due to their biopharmaceutical advantages. However, these particles generally display very weak packing and poor mechanical properties. Hereby, a new methodology is proposed to associate poorly flowing particles to macrostructures targeting the improvement of flowability and redispersibility of the particles.* Cecropia glaziovii*-loaded PLGA microspheres (4.59 ± 0.04 *μ*m) were associated with carrier pellets by film coating in a top-spray fluid bed equipment. Optimal conditions were determined employing a IV-Optimal factorial design and RGB image analysis as 1% (w/v) Kollicoat® Protect as coating polymer (2:1 weight ratio of coating suspension to carrier pellets), containing 5 mg/mL microspheres (loading of 28.07 ± 1.01 mg/g). The method led to an improvement of the overall flowability. No relevant molecular interactions between PLGA microspheres and polymers were found. Microspheres detached rapidly from the surface of the pellets, without agglomeration, when exposed to hydrodynamic forces.* In vitro* release profiles, prior to and after fluid bed coating, showed no relevant changes in drug release rate and extent. The methodology developed is suitable for further applications when an improvement on the flow properties and redispersibility of the product is desired. We showed an easy-to-implement methodology that can be executed without significant increase in costs.

## 1. Introduction

Micro- and nanoparticles have been studied as promising oral drug delivery systems due to their potential biopharmaceutical advantages. Based on their chemical constitutions and granulometric profiles, these systems may enhance drug absorption [1] as well as control drug release [2]. Also, an overall improvement on drug stability may be achieved by micro- and nanoencapsulation of bioactive compounds [3, 4]. On the other hand, these systems face severe technical limitations due to their small particle size, leading to reduced flowability in the solid state. Handling and processing such fine particles may become a relevant industrial problem due to their impaired mechanical properties [5], impacting manufacturing cost and feasibility.

Powder flow may be affected by several characteristics of the particle, such as surface chemistry, texture, contact area, particle density, and hygroscopicity. As a result, adhesion forces (i.e., Van der Waals, capillary, and electrostatic forces) may act on the particles to a larger extent. This is true especially for particles with average diameter below ~10 *μ*m, which are highly subjected to interparticle forces [6, 7]. Due to their reduced particle size, these systems have high surface area, leading to higher contact surface between each individual particle and, therefore, fostering interparticle interactions [7]. Thus, the net interparticle adhesion of the system increases, reducing flowability [8]. For larger particles, on the other hand, gravitational forces are greater than interparticle forces, allowing powders to flow freely [8].

For this reason, industrial scientists are constantly searching for alternatives to improve flowability of micro- and submicrometric particles. One of the most promising strategies is the association of micro/nanoparticles to carrier particles. Over the last decade, an increase in patent applications proposing technologies to solve this issue was noticeable. This is shown in patents claimed by organizations such as 3M Innovative Properties, Co. [9], Applied Nanotech Holdings, Inc. [10], Universidad Nacional Autónoma de México [11], Evonik Röhm Gmbh. [12], and Teva Pharmaceutical Industries, Ltd. [13].

**Figure 1:**
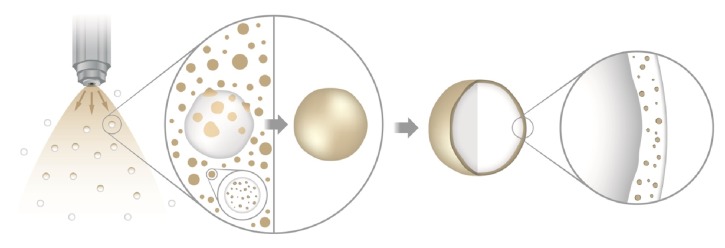
Schematic illustration of the preparation of microspheres-coated pellets by top-spray fluid bed coating.

In this work, we propose a novel standardized methodology to associate poorly flowing particles to macrostructures targeting the improvement of the mechanical properties of the final product. Poly(lactic-co-glycolic acid) (PLGA) microspheres (particle size < 10 *μ*m) were chosen as model particles due to their poor flow properties.* Cecropia glaziovii* freeze-dried extract was employed as model drug due to its high hygroscopicity, which leads to an increase in capillary forces acting on the particles loading it [14].* C. glaziovii* is a highly bioactive vegetal species [15–19] and it has been studied before as a bioactive product for pharmaceutical purposes [20, 21]. In the coating studies, two hydrophilic polymers (Kollidon® VA64 and Kollicoat® Protect) were assessed as coating materials to attach microspheres to the surface of carrier pellets. These polymers were chosen due to their fast-dissolving properties and safety, as they are already used as ingredients of other FDA-approved pharmaceuticals. Also, RGB (Red, Green, and Blue color system) image analysis [22] is proposed as a new and low-cost method to evaluate the total coated surface of pellets. As shown in Figure 1, the methodology studied consisted of the atomization and deposition of microspheres on the surface of carrier pellets using a top-spray fluid bed equipment. The intended structure retained microspheres dispersed homogeneously within the dried polymeric coating, which readily dissolved in contact with aqueous medium.

## 2. Materials and Methods

### 2.1. Materials

Methylene blue and polyvinyl alcohol were purchased from Vetec (Brazil) and Sigma-Aldrich (USA), respectively. Poly(lactic-co-glycolic acid) (PLGA) 503H (50:50 lactic to glycolic acid ratio) was obtained from Boehringer Ingelheim (Germany). Kollidon® VA64 (vinylpyrrolidone-vinyl acetate copolymer) and Kollicoat® Protect (polyvinyl alcohol-polyethylene glycol graft copolymer with polyvinyl alcohol) were kindly donated by BASF (Germany). Commercially-available microcrystalline cellulose 101 pellets (1,000 *μ*m average size) manufactured by extrusion-spheronization (Microcel® Pellets) were donated by Blanver (Brazil).* Cecropia glaziovii* leaves were purchased from the Pluridisciplinary Center of Chemical, Biological, and Agronomic Studies of the University of Campinas (Brazil) and the freeze-dried extract employed as model drug was prepared as previously described in the literature [20]. All other reagents and solvents were of analytical grade.

### 2.2. Preparation of Cecropia glaziovii Extract-Loaded PLGA Microspheres

*Cecropia glaziovii* extract-loaded microspheres were prepared by the double emulsion solvent evaporation/extraction method. Briefly, 12.5 mg of freeze-dried extract was dissolved in 500 *μ*L of sodium hydroxide solution (0.5% w/v) and emulsified with 8 mL of PLGA 503H solution in dichloromethane (2.5 mg/mL) at 13500 rpm for 2 min (Ultra-Turrax® T25Basic, IKA, Germany; dispersing tool S25N-10G) to obtain a water in oil emulsion (w_1_/o). The emulsion was transferred to 80 mL of a solution containing polyvinyl alcohol (0.5% w/v) and sodium chloride (3% w/v) and emulsified at 13,500 rpm for 1 min (dispersing tool S20-25NK-19G) to obtain a w_1_/o/w_2_ double emulsion. The double emulsion was diluted in 100 mL of a polyvinyl alcohol (0.1% w/v) and sodium chloride (3% w/v) solution. The final dispersion was stirred for 2 h to allow the evaporation of dichloromethane and solidification of microspheres. Solidified microspheres were centrifuged (RCF = 3256), washed three times with ultra-pure water, and freeze-dried (LD1500, Terroni, Brazil) for 48 h to obtain a highly-hygroscopic dry powder.

### 2.3. Determination of Coating Variables and Parameters

Two hydrophilic polymers were chosen as coating materials for the association of microspheres to pellets: Kollidon® VA64 and Kollicoat® Protect. Some of the coating conditions were prestandardized experimentally using a top-spray fluid bed equipment (Mini Coater Drier-2, Caleva, UK): 5 g batch, 13 Hz agitation, and air inlet of 12.5 m^3^/s at 50°C. Microspheres (5 mg/mL) were dispersed in the polymer solution containing methylene blue (0.01% w/v). Suspensions were atomized on the surface of pellets (1.5 bar) at a 1.5 mL/min rate. Drying was performed afterwards for 10 min at 60°C.

Three coating parameters were evaluated using a IV-Optimal factorial design (Table 1): (A) coating polymer type, (B) polymer concentration, and (C) coating suspension to pellets ratio (volume to weight). As outcome, total coated surface values were determined by a modified RGB image analysis method [22]. Pellets were spread over a black glass surface and images (200 particles/formulation) were taken using a stereoscope (SZX16, Olympus, Brazil) under controlled light environment at 25 lux (LightMeter, HS1010, China). Photographs were processed using the software ImageJ (1.45s, National Institutes of Health, USA) and color histograms obtained using the plug-in ColorInspector 3D (2.0, FHTW, Germany). RGB frequencies per pixels were transformed into percentage and the total coated surface was estimated by comparison with the RGB profile of uncoated pellets. Pixels corresponding to blue or white colors were considered as “coated” or “uncoated”, respectively.

Regression analysis was carried out employing the software Design-Expert® 8.0.7.1 (State-Ease Inc., USA) and analysis of variance (ANOVA) was applied to identify the significance of single factors, binary interactions, and quadratic factors (*p*<0.05).

### 2.4. Preparation of Microspheres-Coated Pellets

Microspheres (5 mg/mL) were dispersed in the coating polymer as described in Section 3.1. (Kollicoat® Protect 1.0 % w/v at a 2:1 ratio of coating suspension to pellets) without the addition of methylene blue. Three 20 g batches were prepared at 13 Hz agitation with air inlet of 14.0 m^3^/s at 50°C (Mini Coater Drier-2, Caleva, UK). Dispersions were atomized on the surface of the pellets (1.5 bar, 1.5 mL/min) and drying was performed afterwards for 10 min at 60°C. Each batch was coated with three coating layers to achieve a theoretical load of 30 mg of microspheres per g of pellets.

### 2.5. Loading Determination

Microspheres load was determined by a visual counting method using K-Cell® chambers (Intralab, Brazil). A known mass of pellets (n=3) was dispersed in filtered water and agitated by inversion (Tepron, Brazil) for 6 h. Later, 100 *μ*L of the supernatant was collected and transferred to the K-Cell® chamber. The number of particles within the counting area was determined using a light microscope (Bx41, Olympus, USA). Concentration of microspheres was estimated by means of an analytical curve (10 – 750 *μ*g/mL). Concentration values were used to calculate the load of microspheres in pellets (mg/g, n=3).

### 2.6. Attenuated Total Reflectance Fourier Transform Infrared Spectroscopy (ATR-FTIR)

Coating films were carefully extracted from the surface of the pellets using a scalpel. Samples were directly analyzed using a universal diamond ATR accessory coupled to a FTIR spectrophotometer (Frontier, PerkinElmer, USA). Analyses were performed over the range of 4000 to 600 cm^−1^ with a 4 cm^−1^ spectral resolution (32 scans/read).

### 2.7. Determination of Densities and Flow Properties

Flow properties of the products were estimated by means of the Hausner ratio, Carr's index [14, 23], and the static angle of repose [24].

A 100 mL cylinder was filled with a known mass of pellets and the mass to volume ratio was determined as the bulk density (*ρ*b). Samples were submitted to 1250 taps at a tapped density tester (Copley JV1000, UK) and the mass to compacted volume ratio was determined as the tapped density (*ρ*t). Both density values were used to calculate the Hausner ratio (HR; (1)) and Carr's index (CI; (2)). True density values were assessed by helium displacement pycnometry (AccuPycII 1340, Micromeritics Instrument Corp., USA) at a helium flow of 2.2 kg/cm^2^.

(1) HR=ρtρb

(2) CI%=ρt−ρbρt∗100

Static angle of repose was determined by the fixed-base piling method [24]. Samples were filled into a suspended funnel (4 cm height) and discharged to form a conical pile on the ground base. Photographs of the repose piles were analyzed (Size Meter 1.1, LCP/UFSC, Brazil) to determine the angle of repose (n=6).

### 2.8. Cryosection of Coated Pellets

Cross-sections of products were obtained using the microtome-cryostat CM1850UV (Leica Microsystems, Germany). Samples were submerged in freezing medium (Tissue-Tek®, Leica Microsystems, Germany) and frozen in the cryochamber for 2 h at -20°C. Frozen blocks were sectioned at 5 *μ*m thickness using high profile carbon blades (EMSCD500, Leica Microsystems, USA) and analyzed as described in Section 2.9.

### 2.9. Scanning Electron Microscopy (SEM) and Epifluorescence Microscopy (EFM)

Scanning electron microscopy was performed on a JSM-6390LV microscope (JEOL, USA) using an accelerating voltage of 10 kV. Samples were coated with a fine gold layer before SEM. Fluorescence analyses were performed using an epifluorescence microscope (Bx41, Olympus, USA) equipped with a red filter (excitation = 510 – 550 nm, emission = 590 nm).

### 2.10. Assessment of Mechanical Properties of Coating Films

Mechanical properties of coating films were assessed by a texture analyzer (TA.HD Plus, Stable MicroSystems, UK) equipped with a 300 N loading cell. Percentage of elongation at breakpoint was determined according to the ASTM standard method D882 [25]. Coating films were carefully extracted from the surface of coated pellets using a scalpel (n=5), placed on the extension grips of the equipment, and stretched uniaxially (50 mm/min) until breakage occurred.

### 2.11. Microspheres Detachment from Pellets and Subsequent Disaggregation

Detachment of microspheres from the surface of pellets and their subsequent disaggregation in the aqueous medium were evaluated using the reciprocating cylinder apparatus BioDis RRT10 (Erweka, Germany) (n=6). 250 mL 0.1 N HCl solution (pH 1.2) at 37°C was used as medium. Pellets (1 g) were retained within the dipping tubes by 50 *μ*m meshes and submitted to a total of 20 dips into the aqueous medium. Every two immersions, an aliquot was collected to determine the concentration of microspheres detached from the pellets using K-Cell® chambers (Section 2.5) and their average particle size by laser diffraction (Section 2.12).

### 2.12. Laser Diffraction Particle Size Analysis

Particle size distribution was determined using the MasterSizer 2000 laser diffraction equipment (Malvern, UK) with an aqueous sample dispersion unit (Hydro 2000SM, Malvern, UK). Results were calculated by the Fraunhofer diffraction model and the equivalent volume average diameters (D_4,3_) were determined (n=20). Specific surface area was calculated using the true density of the particles (Section 2.7).

### 2.13. Release Studies

Drug release profiles were obtained using the paddle method (USP-2 Apparatus model 299, Nova Ética, Brazil) at 75 rpm. The dissolution medium was phosphate buffer solution (900 mL, pH 6.8) at 37°C. Microspheres-coated pellets and freeze-dried microspheres were placed at the bottom of the dissolution vessels (n=6) and aliquots were collected at defined time intervals. Samples were centrifuged and analyzed by high pressure liquid chromatography (HPLC) to determine the cumulative percentage release of the model drug (Section 2.14.). Profiles were compared employing the similarity factor (f_2_, DDSolver software) [26].

### 2.14. HPLC Analysis

Chromatographic analyses were performed to quantify chlorogenic acid (CGA), a chemical marker of* C. glaziovii* extracts. A previously validated method [20] was employed using a PerkinElmer Series 200 chromatographer. Stationary phase was a Luna C18(2) column (5 *μ*m, 150 x 4.6 mm, 100 Å; Phenomenex, USA). Mobile phase consisted of acetonitrile and trifluoroacetic acid 0.05% (v/v) at a proportion of 11:89 (1 mL/min). Samples were injected in duplicate (20 *μ*L) and quantification was performed at 320 nm by comparison with a CGA primary standard curve (1 – 200 *μ*g/mL).

## 3. Results and Discussion

### 3.1. Determination of Optimal Coating Variables

*C. glaziovii*-loaded PLGA microspheres showed mean particle size (D_4,3_) of 4.59 ± 0.04 *μ*m and specific surface area of 1.85 ± 0.01 m^2^/g. Based on the particle size distribution, these microspheres are expected to display impaired flowability, as confirmed by the angle of repose, Hausner ratio, and Carr's index (Section 3.4).

A fraction of the microcrystalline cellulose pellets (850 – 1000 *μ*m) was separated by sieving the commercially-available material and it had a weight yield of 74 ± 2%. This fraction, named “carrier pellets”, was employed in further experiments to reduce the influence of the pellet size distribution on the coating outcome, as a monodisperse material leads to higher homogeneity in thickness and coverage of coating layers [27].

Coating studies were performed employing a IV-Optimal factorial design, combining two polymers in three concentrations (Table 2). Within the experimental domain, total coated surface values within the range of 20.0 to 99.1% were obtained. RGB analysis has proven to be adequate for assessing the total coated surface when colored coatings are employed. This technique was used as a research tool in food science in the past [22], but it has not been applied to coating analyses in pharmaceuticals as far as the authors are aware. Coating suspensions containing methylene blue presented RGB histograms with increasing frequencies of blue as the coated surface increased. As shown in Supplementary Figure S1 (supplementary materials), RGB histograms obtained for coated formulations are very distinct when compared with carrier pellets (uncoated), allowing the quantification of coated surface by differential comparison of RGB pixel frequencies.

ANOVA results showed factors A (polymer type;* p*=0.0014), B (polymer concentration;* p*=0.0004), and C (coating suspension to pellets ratio;* p*<0.0001) influenced the total coated surface. Also, interactions AB (*p*=0.0282) and BC (*p*=0.0023) were statistically significant, indicating a dependent behavior between polymer type and concentration (interaction AB) and also between polymer concentration and coating suspension to pellets ratio (interaction BC). The mathematical model describing this response was statistically significant (*p*<0.0001). R^2^ (0.9914), adjusted R^2^ (0.9795), and predictive R^2^ (0.9484) of the model were excellent indicatives of the agreement of the experimental to the theoretical dataset. The equation describing the response as a function of the relevant coded terms and their coefficients is

(3) Coated  surface%=62.49−3.91A+8.50B1−0.52B2−36.24C1+10.21C2+3.18AB1−3.97AB2−7.39B1C1+2.58B2C1+8.24B1C2+3.02B2C2

**Figure 2:**
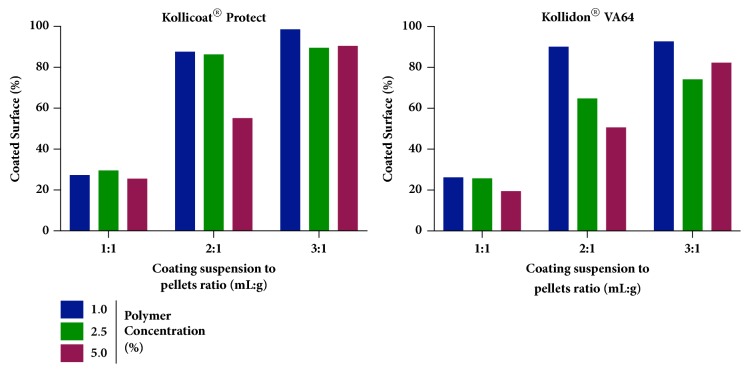
Bar graphs correlating the percentage of coated surface to polymer type, polymer concentration, and coating suspension to pellets ratio.

In general, higher percentages of coated surfaces were found for Kollicoat® Protect when compared with Kollidon® VA64 (Figure 2). This effect could be associated with the viscosity of each polymer solution, as it may cause a negative effect on the coating efficiency [28]. Nevertheless, the coefficients for all terms involving polymer type were not high when compared with the other terms (3).

On the other hand, polymer concentration displayed remarkable influence on the response. Lower percentages of coated surfaces were found as the polymer concentration increased (Figure 2). In this case, coating suspension droplets formed during atomization have increased size as the concentration of the polymer increases due to an increase of the overall viscosity of the solution [29]. Consequently, the spreadability of the droplets reduces, leading to lower surface coverage.

When a ratio of 3:1 (coating suspension (mL) to pellets (g)) was employed, the effect of viscosity was null due to the dilution of the polymer and the increased volume of coating suspension available to cover the entire surface of the carrier pellets. Changing the ratio to 2:1 or 1:1 led to a reduction of the total coated surface, as there was not enough coating material to cover the entire surface area of the pellets. In summary, Figure 2 shows the best coating conditions (coated surface > 90%) were obtained employing low polymer concentration (1%) and a 2:1 or 3:1 (mL/g) coating suspension to pellets ratio. Although both ratios showed similar results, the 2:1 ratio is a more viable option due to the reduction of the coating time, decreasing the total processing cost. Kollicoat® Protect seems to be a smarter polymer choice due to its low moisture-permeability properties, high water solubility, biocompatibility, reduced need for plasticizer, and increased mechanical strength [30, 31].

### 3.2. Attenuated Total Reflectance Fourier Transform Infrared Spectroscopy (ATR-FTIR)

**Figure 3:**
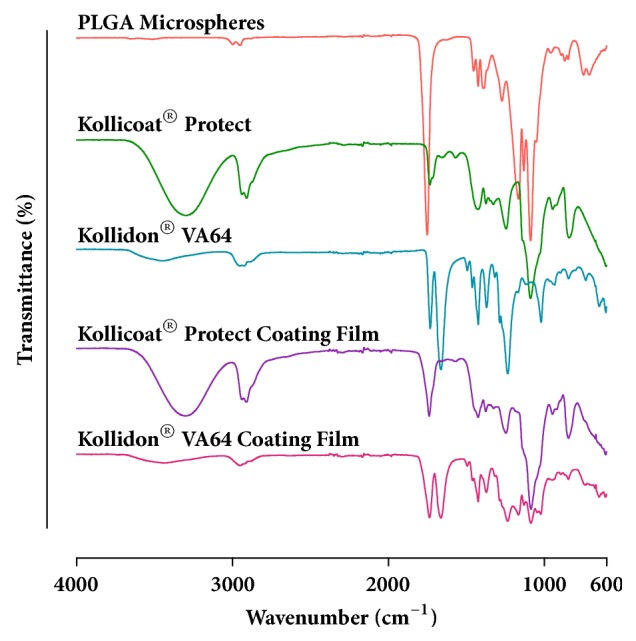
ATR-FTIR spectra of blank PLGA microspheres (without* C. glaziovii* extract), polymers, and coating films containing blank PLGA microspheres.

Molecular interactions between blank PLGA microspheres and the coating polymers were monitored by ATR-FTIR. This assessment is especially relevant due to the impact molecular interactions may have on the mechanical properties of polymers [27]. Products containing* C. glaziovii* extract were not assessed due to their chemical complexity, which did not allow proper and conclusive analyses of ATR-FTIR spectra. Nonetheless, a representative* C. glaziovii* extract spectrum is provided as Supplementary Figure S2. PLGA microspheres' spectrum showed weak OH stretching (3700 – 3300 cm^−1^) and -CH stretching (3000 – 2900 cm^−1^) bands. It also showed strong bands for C=O (1751.6 cm^−1^) and C-O stretching (1500 – 1000 cm^−1^), which refer to the lactic-co-glycolic acid chains of PLGA. Dislocation or disappearance of characteristic bands for PLGA microspheres were not noticed on the coating films spectra (Figure 3). In fact, for both polymers, the spectra corresponded merely to the overlapping of each of the components' spectra, indicating that no relevant molecular interactions occurred between microspheres and coating polymers during fluid bed coating. This indicates that the attachment mechanism by which the particles remained associated with the coating film is merely physical, caused by the intercalation and entrapment of the microspheres within the polymeric layers.

### 3.3. Microspheres-Coated Pellets

Optimal coating conditions for adequate surface coverage were determined as 1% (w/v) Kollicoat® Protect and a suspension to pellets ratio of 2:1 (mL/g), as described in Section 3.1. Formulations were prepared under these conditions with a total of three coating layers. The process yield, determined as the percentage of the experimental to the theoretical mass after coating, was 93.7 ± 1.2%. The load of microspheres on the surface of pellets was 28.07 ± 1.01 mg/g. The yield of the formulation in terms of load of microspheres was 95.6 ± 3.4%, which is in agreement with the overall yield of the process.

### 3.4. Micromorphological Analysis

**Figure 4:**
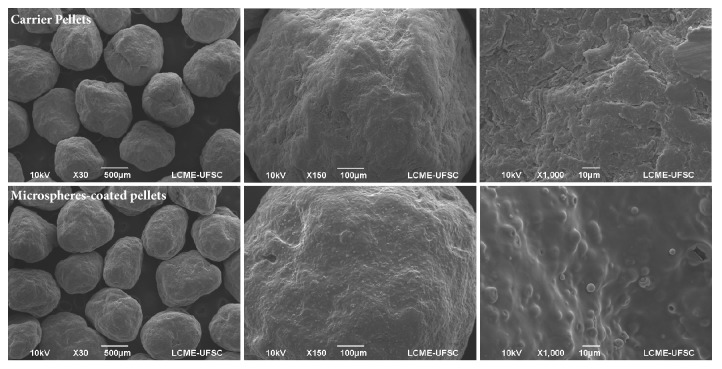
SEM micrographs of carrier pellets (**first row**) and microspheres-coated pellets (**second row**) at 30, 150, and 1000x magnification (first, second, and third column, respectively).

SEM of microspheres exhibited spherical structures, with particle size in agreement with the results obtained by laser diffraction (Supplementary Figure S3). Carrier pellets (Figure 4, first line) presented morphological characteristics in agreement with the expected for typical microcrystalline cellulose pellets. The rough structure observed resulted from the superposition of the microcrystalline cellulose particles during the extrusion-spheronization process [32].

**Figure 5:**
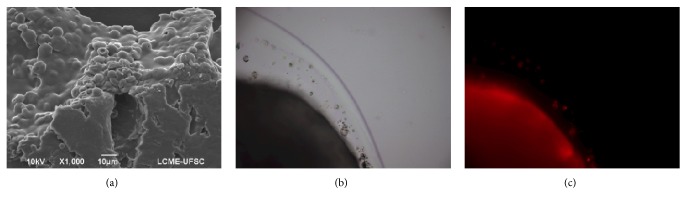
(a) SEM micrograph of the cryosectioned microspheres-coated pellets (1000x). Micrographs of the cryosectioned microspheres-coated pellets under (b) light and (c) epifluorescence microscopy (40x magnification).

On the other hand, microspheres-coated pellets showed a smoother surface due to the presence of the coating polymer. PLGA microspheres homogeneously dispersed in the polymeric coating film were also observed (Figure 4, second line). Cryosection images confirmed microspheres were dispersed on different layers within the polymeric coating (Figure 5). This homogeneous dispersion was a strong indication that agglomeration did not occur during the coating process, and the polymer concentration was adequate to maintain individual microparticles separated from each other after drying. Such findings are relevant as the drying process leads to the shrinkage of the coating layer as water is removed, reducing the distance between the dispersed particles. If the interparticle distance is too short (if not enough polymer is used), particles may agglomerate, potentially impairing their ability to be released from the coating film in a dispersed state.

Epifluorescence microscopy (EFM) showed* C. glaziovii* freeze-dried extract particles displayed intense fluorescence signal (Supplementary Figure S4), which can be attributed to the high content of phenolic compounds of the extract [33]. Consequently,* C. glaziovii*-loaded PLGA microspheres also displayed fluorescence signal under EFM. Cryosectioned pellets clearly displayed the polymeric layer around the microcrystalline cellulose core (Figure 5(b)), with fluorescent particles dispersed within it (Figure 5(c)). Also, a disperse fluorescence signal was noticed in parts of the polymeric film. It is hypothesized that this could be caused by the early release of the extract from the microspheres during the coating process. This effect may be attributed to the coating process itself, as the microspheres are maintained under constant stirring in aqueous medium, potentially fostering partial release of the extract. As the coating layer dried, the extract also solidified, potentially causing the diffuse fluorescent signal observed. A set of EFM micrographs is provided as supplementary data (Supplementary Figures S5 and S6).

### 3.5. Densities and Flowability

A threefold decrease was noticed on the average bulk density when comparing the freeze-dried extract with extract-loaded PLGA microspheres (*p*<0.0001, Table 3). The reduction on bulk density means an equal mass of the product will occupy a higher volume, therefore reducing the total dose that can be compressed into tablets or encapsulated in gelatin capsules. In general, reduction of the total dose is undesired, especially when high dosages are needed to achieve drug therapeutic concentrations in blood. On the other hand, a high bulk density was found for the microspheres-coated pellets. No significant differences between carrier and coated pellets were noticed (*p*>0.05).

Regarding tapped density, a similar behavior was noticed.* C. glaziovii* extract had an increase in density after compaction of around 30%. Meanwhile, extract-loaded microspheres displayed an increase in density of around 90%. This difference is indicative of the increased interparticle forces that act on the microstructured system. These forces are responsible for maintaining the individual particles separated from each other, reducing the bulk density and increasing the difference between bulk and tapped densities of the material [7]. Carrier and coated pellets showed no relevant differences between their bulk and tapped densities.

True density results showed* C. glaziovii* extract has a denser particle structure when compared with* C. glaziovii*-loaded microspheres. Microspheres-coated pellets also showed denser structure when compared to their uncoated form due to the addition of the polymeric coating layer, which increases the density and decreases the porous network of the system.

Hausner ratio and Carr's index values were assessed as indicative of the flowability of the products. As previously discussed, the difference between these densities is related to the interparticle forces acting on the particulate system [7, 8]. Since it is known that the interparticle forces also influence flow, these density values can be used to estimate the flowability of the products. Freeze-dried* C. glaziovii* extract showed unsatisfactory results for both parameters, indicating poor flowability. The encapsulation of the extract in PLGA microspheres, though advantageous in many biopharmaceutical aspects, led to a product with even poorer flowability. The reduction in flowability was directly related to the smaller particle size of the microspheres when compared with the original particle size of the freeze-dried extract (Supplementary Figure S4). On the other hand, carrier pellets, as well as microspheres-coated pellets, showed excellent indexes, indicating free-flowing properties.

**Figure 6:**
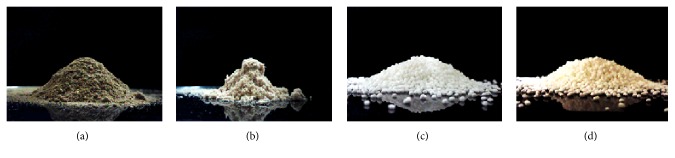
Repose piles obtained for the (a)* C. glaziovii* extract, (b)* C. glaziovii-*loaded PLGA microspheres, (c) carrier pellets, and (d) microspheres-coated pellets.

The angle of repose, which is a much more reliable indicative measure of flowability, showed similar results.* C. glaziovii* extract (37.8 ± 1.4°) and microspheres (48.4 ± 1.3°) were characterized as poor-flowing powders. The repose pile formed by the microspheres displayed an irregular shape (Figure 6(b)), characteristic of products exhibiting extreme resistance to flow. In contrast, microspheres-coated pellets showed free-flowing properties (14.5 ± 0.3°) and a broad repose pile (Figure 6(d)). Therefore, it is concluded that the association of poorly flowing microspheres to pellets is a great alternative towards providing free-flow to the product.

### 3.6. Mechanical Properties of the Coating Films

Texture analyses of the coating films, with and without microspheres, showed that the incorporation of microspheres to the coating solution led to a less flexible film (*p*<0.0001). The elongation at breakpoint for Kollicoat® Protect film obtained under optimized conditions but without microspheres was 37.4 ± 2.5%. Meanwhile, the elongation at breakpoint for the microspheres-coated pellets' film was only 21.5 ± 3.2%. The elongation at breakpoint is an important parameter regarding flexibility of films because it is indicative of the ability of the film to deform before breaking. The addition of microspheres to the coating solution led to the formation of a weaker and less flexible coating, likely due to the discontinuity of the coating layers, caused by the interposition of the particles within. This result is corroborated by the ATR-FTIR results (Section 3.2.), which indicated that no molecular interactions occurred between the polymeric film and PLGA microspheres. If strong molecular interactions took place, a different mechanical behavior would be more likely, giving rise to a stronger film, with potentially higher elongation at breakpoint.

### 3.7. Microspheres Detachment from Pellets and Subsequent Disaggregation

**Figure 7:**
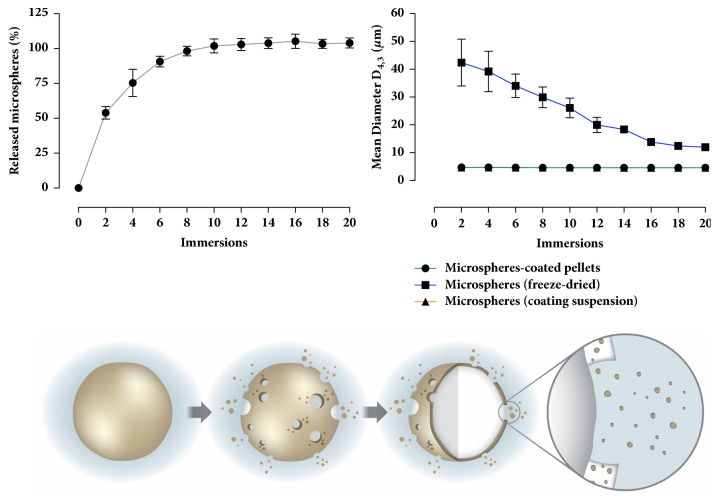
(**Top**) Plots correlating the number of immersions to the percentage of detached microspheres from the coated pellets (left graph) and to the average diameter of the particles (right graph; note: lines for microspheres-coated pellets and suspended microspheres overlapped). (**Bottom**) Schematic representation of the behavior of microspheres-coated pellets in contact with aqueous media. Coating polymer readily dissolves releasing disaggregated microspheres.

Detachment of microspheres from the surface of the coated pellets was assessed using a reciprocating cylinder apparatus. In general, detachment occurred quickly (Figure 7, top left graph). It is assumed that the polymer readily dissolved in contact with the aqueous medium, releasing the particles. Laser diffraction analysis showed microspheres immediately detached back to their original average particle size when compared with the coating suspension prior to fluid bed coating (Figure 7, top right graph). On the other hand, freeze-dried microspheres (prior to redispersion and coating) took much longer to disaggregate when compared with those microspheres detached from the coated pellets. The instantaneous disaggregation of the particles is a result of their homogeneous disperse state in the polymeric coating film, as shown by the SEM micrographs (Figures 4 and 5(a)). When the polymer dissolved, particles instantly detached back to their original particle size, as shown by the schematic representation in Figure 7 (bottom). The aforementioned results showcase the potential of this technique in improving the redispersibility of polymeric microspheres.

### 3.8. Release Studies

**Figure 8:**
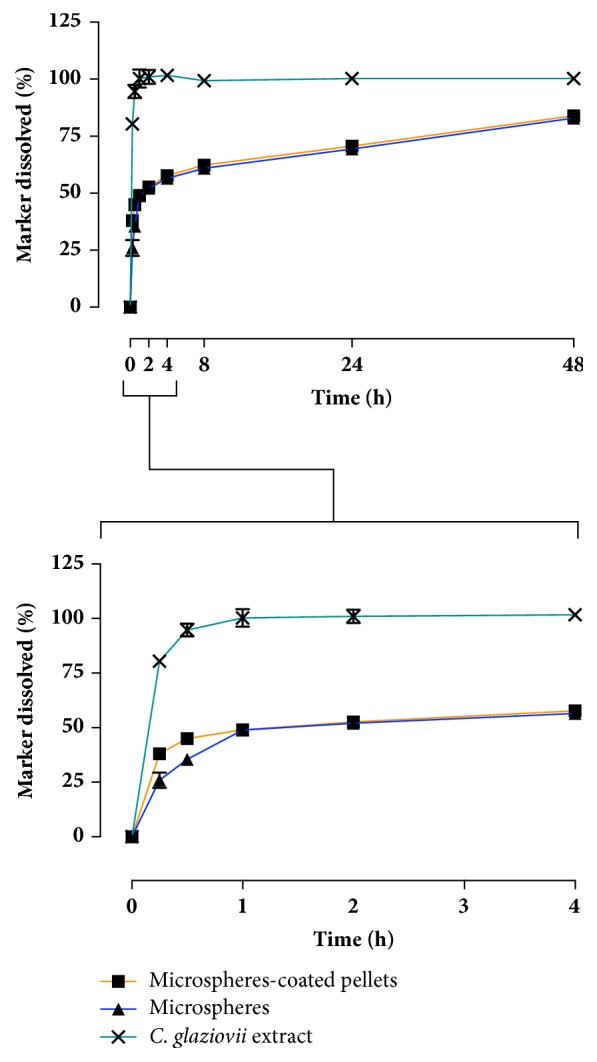
*In vitro* chlorogenic acid (chemical marker) release profiles from microspheres, microspheres-loaded pellets, and freeze-dried* C. glaziovii* extract.

*In vitro* release studies were carried out to verify if the attachment of PLGA microspheres to the surface of pellets by fluid bed coating led to relevant changes in drug release profile. Chlorogenic acid, a major constituent of* C. glaziovii* extracts, was monitored by HPLC to determine the cumulative release of the marker from the microspheres.  Figure 8 shows the release profiles obtained. It is noticeable that the microspheres themselves were able to prolong the release of chlorogenic acid when compared with the dissolution profile of the freeze-dried extract (similarity factor f_2_ = 7).

Release profiles of the chemical marker from microspheres prior to and after the fluid bed coating showed very similar rate and extent (f_2_ = 48). However, microspheres released from pellets had a slightly increased burst release effect when compared with unprocessed microspheres. The burst release could be related to the early release of the vegetal extract in the coating suspension during fluid bed processing. As shown by the EFM micrographs (Figure 5(c)), it seems that a small amount of free extract was dispersed in the polymer coating layer, outside of the microspheres. When the polymer dissolved, the free extract may dissolve as well, leading to the pronounced burst release effect observed. Faster disaggregation of microspheres after detachment from the surface of the pellets can also contribute towards the burst release effect by allowing faster release of the model drug entrapped.

### 3.9. Comparison with Other Strategies to Promote Flow Enhancement

Promoting flow enhancement to micrometric and submicrometric particles is of high importance for industrial applications. The general understanding in the industrial pharmacy field is that transforming small particles into multiparticulate systems (e.g., wet/dry granulation, extrusion-spheronization) is the most viable alternative to solve flowability issues [34]. These strategies are valuable for regular powder mixtures but may be inadequate for multifunctional particles such as drug-loaded microspheres. Under these situations, alternative methods such as the proposed herein can be much more advantageous.

For instance, a modified pellet formulation has been proposed before containing an inner matrix layer of nanoparticles [12]. Potentially, the large particle size of pellets can enhance flowability. However, the inner compartment is likely to impact the product performance of the nanoparticles, and therefore it would not be appropriate for a “fast-release” system. Following similar principles, a proprietary technique to associate two particle populations (one nanosized and another microsized) has been proposed [9]. Also, a variety of nanocomposite systems have been suggested. Clay and carbon nanotubes have been successfully transformed into nanocomposites, where nanoparticles were coated onto the surface of polymeric spheres by ball-milling process [10]. A drug delivery vehicle comprising a carrier particle bearing drug microparticles on its surface has also been investigated [13], whereas the deposition technology relied on sublimation.

More applicable techniques such as spray drying and electrostatic coating have also displayed promising results for this application. For instance, spray drying of amorphous drug nanoparticles with lactose generated a matrix-like structure that displayed fast-dissolving properties [35]. Alternatively, core-shell-like structures can be obtained by spray drying drug particles concurrently with silica particles [35]. The manufacturing of redispersible granules containing TiO_2_ nanoparticles has also been described using the spray drying technique, leading to stable 20 to 50 *μ*m spherical granules [36]. TiO_2_ nanoparticles have also been associated with the surface of plastic granules using a combination of an electrostatic adhesion and heat treatments [37].

Nonetheless, as far as the authors are concerned, none of these current technologies are as simple and easy to implement as the standard method developed in this study. Pelletization and wet/dry granulation technologies are too aggressive on the nanoparticle's structure and often lead to products that do not disintegrate and/or release their contents as fast as would be desirable. Spray drying may lead to particulate products that are still too small to display optimal flow properties. Therefore, the top-spray polymeric coating on carrier pellets technology as proposed here can be an ideal alternative to combine nano/microstructures with larger particles (millimetric range) with the goal of improving flowability and redispersibility without impairing product performance.

## 4. Conclusion

A novel approach with the goal of improving the flow properties and redispersibility of micro- and submicrometric particles was assessed, targeting the production of an industrially-viable particulate product. Poorly flowing PLGA microspheres containing a model vegetal extract were successfully associated with pellets by fluid bed coating employing the polymer Kollicoat® Protect. An excellent improvement of the flow properties of the product was achieved. Microspheres readily detached from the surface of the pellets into the aqueous medium, without aggregation and with minimal interference on the release profile (rate and extent) of the chemical marker.

The methodology proposed is suitable for the association of different types of micrometric and submicrometric particles when an improvement of the overall flowability and redispersibility is desired. Based on our results, we expect this methodology can be expanded to other systems besides microspheres (e.g., polymeric nanoparticles). This approach is considered easily scalable and economically-viable as the equipment needed for fluid bed coating is already used in the manufacture of solid dosage forms, potentially reducing implementation costs. The association of nano/microstructures to pellets may become a viable solution to common issues related to the lack of flow and redispersibility in dried pharmaceutical particulate systems.

## Figures and Tables

**Table 1:** Experimental domain evaluated (IV-Optimal factorial design).

Factor	Levels		
	-1	0	+1
(A) Polymer type	Kollicoat® Protect	-	Kollidon® VA64
(B) Polymer concentration (% w/v)	1.0	2.5	5.0
(C) Coating suspension to pellets ratio (mL/g)	1:1	2:1	3:1

**Table 2:** Total coated surface values as a function of polymer type, concentration, and ratio.

#	Factors			Total coated surface (%)
	(A) Polymer type	(B) Polymer concentration (% w/v)	(C) Coating suspension to pellets ratio (mL/g)	
1	Kollidon® VA64	2.5	1:1	26.3
2	Kollidon® VA64	2.5	2:1	65.4
3	Kollidon® VA64	1.0	1:1	26.8
4	Kollidon® VA64	5.0	3:1	82.9
5	Kollicoat® Protect	2.5	2:1	86.9
6	Kollicoat® Protect	2.5	1:1	30.3
7	Kollidon® VA64	5.0	1:1	20.0
8	Kollicoat® Protect	5.0	1:1	26.1
9	Kollidon® VA64	2.5	2:1	63.1
10	Kollicoat® Protect	5.0	2:1	55.7
11	Kollicoat® Protect	2.5	2:1	85.2
12	Kollicoat® Protect	1.0	2:1	88.2
13	Kollidon® VA64	1.0	3:1	93.3
14	Kollidon® VA64	5.0	2:1	51.2
15	Kollicoat® Protect	1.0	3:1	99.1
16	Kollicoat® Protect	2.5	3:1	90.1
17	Kollidon® VA64	1.0	2:1	90.7
18	Kollicoat® Protect	1.0	1:1	27.9
19	Kollicoat® Protect	5.0	3:1	91.0
20	Kollidon® VA64	2.5	3:1	74.7

**Table 3:** Densities and flow-indicative parameters of the products.

Parameters	*C. glaziovii* extract	Microspheres	Carrier pellets	Coated pellets
Bulk density (g/cm^3^)	0.31 ± 0.01	0.11 ± 0.01	0.69 ± 0.02	0.74 ± 0.03
Tapped density (g/cm^3^)	0.40 ± 0.02	0.21 ± 0.01	0.74 ± 0.01	0.78 ± 0.02
True density (g/cm^3^)	1.564 ± 0.002	1.461 ± 0.001	1.499 ± 0.001	1.571 ± 0.005
Hausner ratio	1.27 ± 0.02	1.81 ± 0.01	1.05 ± 0.01	1.03 ± 0.01
Carr's index (%)	21.0 ± 1.4	44.7 ± 0.2	5.3 ± 1.0	11.8 ± 0.8
Angle of repose (°)	37.8 ± 1.4	48.4 ± 1.3	20.9 ± 0.6	14.5 ± 0.3

## Supplementary Materials

Supplementary Materials Supplementary Materials provided online include 6 supplementary figures. RGB color histograms of uncoated and coated pellets (Supplementary Figure S1). Representative ATR-FTIR spectrum of* C. glaziovii* freeze-dried extract (Supplementary Figure S2). Scanning electron micrograph of* C. glaziovii *microspheres (Supplementary Figure S3). Light and epifluorescence micrographs of* C. glaziovii *extract (Supplementary Figure S4). Cross-sectioned microspheres-coated pellets (Supplementary Figures S5 and S6).

## References

[1] Makhlof A., Tozuka Y., Takeuchi H. (2011). Design and evaluation of novel pH-sensitive chitosan nanoparticles for oral insulin delivery. *European Journal of Pharmaceutical Sciences*.

[2] Velasquez A. A., Ferreira L. M., Stangarlin M. F. L., Da Silva C. D. B., Rolim C. M. B., Cruz L. (2014). Novel Pullulan–Eudragit® S100 blend microparticles for oral delivery of risedronate: Formulation, in vitro evaluation and tableting of blend microparticles. *Materials Science and Engineering C: Materials for Biological Applications*.

[3] Tyagi L. K., Kori M. L. (2014). Stability study and *in-vivo* evaluation of lornoxicam loaded ethyl cellulose microspheres. *International Journal of Pharmaceutical Sciences and Drug Research*.

[4] Li N., Kommireddy D. S., Lvov Y., Liebenberg W., Tiedt L. R., De Villiers M. M. (2006). Nanoparticle multilayers: Surface modification of photosensitive drug microparticles for increased stability and in vitro bioavailability. *Journal of Nanoscience and Nanotechnology*.

[5] Yang J., Sliva A., Banerjee A., Dave R. N., Pfeffer R. (2005). Dry particle coating for improving the flowability of cohesive powders. *Powder Technology*.

[6] Hickey A. J., Crowder T. M., Louey M. D., Orr N. (2003). *A Guide to Pharmaceutical Particulate Science*.

[7] Crowder T. M., Hickey A. J. (2000). The physics of powder flow: Applied to pharmaceutical solids. *Pharmaceutical Technology*.

[8] Li Q., Rudolph V., Weigl B., Earl A. (2004). Interparticle van der Waals force in powder flowability and compactibility. *International Journal of Pharmaceutics*.

[9] Shinbach M. P., Baran J. R. (2008). Method of making compositions including particles. *3M Innovative Properties, US20080152913A11*.

[10] Mao D., Yaniv Z. (2010). Composites. *Applied Nanotech Holdings, WO2010104710A1*.

[11] Quintanar G. D., Ganem R. F. A., Camacho O. E. A. (2011). Pharmaceutical coating based on a mixture of solid lipidic nanoparticles and polymers. *UNAM Universidad Nacional Autónoma de Mexico, MX2010005803A*.

[12] Lizio R., Petereit H.-U., Trupti D., Gottschalk M. (2013). Multiparticulate form of administration, comprising nucleic acid-containing mucoadhesive active ingredients, and method for producing said form of administration. *Evonik Röhm Gmbh, US8568778B2*.

[13] Lerner E. I., Rosenberger V., Flashner-Barak M., Drabkin A., Moldavski N. (2014). Drug microparticles, processes of preparing them and a drug delivery vehicle comprising them. *Teva Pharmaceutical Industries Ltd, US8663703B2*.

[14] Emery E., Oliver J., Pugsley T., Sharma J., Zhou J. (2009). Flowability of moist pharmaceutical powders. *Powder Technology*.

[15] Arend D. P., dos Santos T. C., Cazarolli L. H. (2015). *In vivo* potential hypoglycemic and *in vitro* vasorelaxant effects of* Cecropia glaziovii* standardized extracts. *Brazilian Journal of Pharmacognosy*.

[16] Delarcina S., Lima-Landman M. T. R., Souccar C., Cysneiros R. M., Tanae M. M., Lapa A. J. (2007). Inhibition of histamine-induced bronchospasm in guinea-pigs treated with *Cecropia glaziovii* Sneth. extracts and correlation with the *in vitro* activity in tracheal muscles. *Phytomedicine*.

[17] Ninahuaman M. F. M. L., Souccar C., Lapa A. J., Lima-Landman M. T. R. (2007). ACE activity during the hypotension produced by standardized aqueous extract of *Cecropia glaziovii* Sneth: A comparative study to captopril effects in rats. *Phytomedicine*.

[18] Rocha F. F., Lapa A. J., De Lima T. C. M. (2002). Evaluation of the anxiolytic-like effects of *Cecropia glaziovi* Sneth. in mice. *Pharmacology Biochemistry & Behavior*.

[19] Rocha F. F., Lima-Landman M. T. R., Souccar C., Tanae M. M., De Lima T. C. M., Lapa A. J. (2007). Antidepressant-like effect of *Cecropia glazioui* Sneth. and its constituints - *In vivo* and *in vitro* characterization of the underlying mechanism. *Phytomedicine*.

[20] Beringhs A. O., Dalmina M., Creczynski-Pasa T. B., Sonaglio D. (2015). Response Surface Methodology IV-Optimal design applied to the performance improvement of an RP-HPLC-UV method for the quantification of phenolic acids in *Cecropia glaziovii* products. *Brazilian Journal of Pharmacognosy*.

[21] Beringhs A. O., Souza F. M., de Campos A. M., Ferraz H. G., Sonaglio D. (2013). Technological development of *Cecropia glaziovi* Snetl. extract pellets by extrusion-spheronization. *Brazilian Journal of Pharmacognosy*.

[22] Foca G., Masino F., Antonelli A., Ulrici A. (2011). Prediction of compositional and sensory characteristics using RGB digital images and multivariate calibration techniques. *Analytica Chimica Acta*.

[23] Carr R. L. (1965). Evaluating flow properties of solids. *Chemical Engineering*.

[24] Wang W., Zhang J., Yang S., Zhang H., Yang H., Yue G. (2010). Experimental study on the angle of repose of pulverized coal. *Particuology*.

[25] ASTM (2002). Standard test method for tensile properties of thin plastic sheeting. D882. *Annual Book of American Standard Testing Methods*.

[26] Zhang Y., Huo M., Zhou J. (2010). DDSolver: An add-in program for modeling and comparison of drug dissolution profiles. *The AAPS Journal*.

[27] McGinity J. W., Felton L. A. (2008). *Aqueous Polymeric Coatings for Pharmaceutical Dosage Forms*.

[28] Teunou E., Poncelet D. (2002). Batch and continuous fluid bed coating - Review and state of the art. *Journal of Food Engineering*.

[29] Hede P. D., Bach P., Jensen A. D. (2008). Top-spray fluid bed coating: Scale-up in terms of relative droplet size and drying force. *Powder Technology*.

[30] Joshi S., Petereit H.-U. (2013). Film coatings for taste masking and moisture protection. *International Journal of Pharmaceutics*.

[31] Preis M., Woertz C., Schneider K. (2014). Design and evaluation of bilayered buccal film preparations for local administration of lidocaine hydrochloride. *European Journal of Pharmaceutics and Biopharmaceutics*.

[32] Dreu R., Širca J., Pintye-Hodi K., Burjan T., Planinšek O., Srčič S. (2005). Physicochemical properties of granulating liquids and their influence on microcrystalline cellulose pellets obtained by extrusion-spheronisation technology. *International Journal of Pharmaceutics*.

[33] López M., Martínez F., Del Valle C., Ferrit M., Luque R. (2003). Study of phenolic compounds as natural antioxidants by a fluorescence method. *Talanta*.

[34] Shanmugam S. (2015). Granulation techniques and technologies: Recent progresses. *BioImpacts*.

[35] Melzig S., Niedbalka D., Schilde C., Kwade A. (2018). Spray drying of amorphous ibuprofen nanoparticles for the production of granules with enhanced drug release. *Colloids and Surfaces A: Physicochemical and Engineering Aspects*.

[36] Faure B., Lindelov J. S., Wahlberg M., Adkins N., Jackson P., Bergstrom L. (2010). Spray drying of TiO_2_ nanoparticles into redispersible granules. *Powder Technology*.

[37] Sutisna, Rokhmat M., Wibowo E., Khairurrijal, Abdullah M. (2017). Coating TiO_2_ nanoparticles on the surface of transparent plastic granules using combined electrostatic and heating methods for the photocatalytic degradation of organic pollutants in water. *Environmental Nanotechnology, Monitoring and Management*.

