# Supplementary material for: Association of PLGA Microspheres to Carrier Pellets by Fluid Bed Coating: A Novel Approach towards Improving the Flowability of Microparticles
Source: J Pharm (Cairo). 2018 Jul 2;2018:3874348. doi: 10.1155/2018/3874348 (PMC6051010; doi:10.1155/2018/3874348)
Supplement: Supplementary Materials — provided online include 6 supplementary figures. RGB color histograms of uncoated and coated pellets (Supplementary Figure S1). Representative ATR-FTIR spectrum of C. glaziovii freeze-dried extract (Supplementary Figure S2). Scanning electron micrograph of C. glaziovii microspheres (Supplementary Figure S3). Light and epifluorescence micrographs of C. glaziovii extract (Supplementary Figure S4). Cross-sectioned microspheres-coated pellets (Supplementary Figures S5 and S6). [file 3874348.f1.pdf]

Journal of Pharmaceutics

Association of PLGA microspheres to carrier pellets by fluid bed coating: a novel approach towards improving the flowability of microparticles

André O'Reilly Beringhs[a,b], Aline Benedita dos Santos Fonseca[a], Angela Machado De Campos[a], Diva Sonaglio[a]

[a] *Department of Pharmaceutical Sciences, Center of Health Sciences, Federal University of Santa Catarina, Florianópolis, Brazil.*

[b] *Department of Pharmaceutical Sciences, School of Pharmacy, University of Connecticut, Storrs, United States of America.*

SUPPLEMENTARY MATERIALS

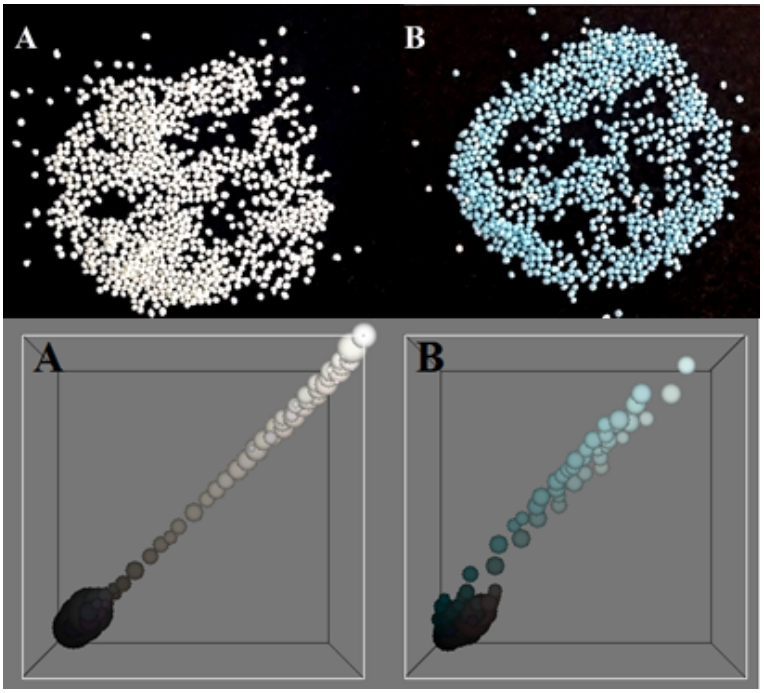

Figure S1: RGB color histograms obtained for (**A**) uncoated carrier pellets and (**B**) experiment #15 formulation (99.1% of coated surface).

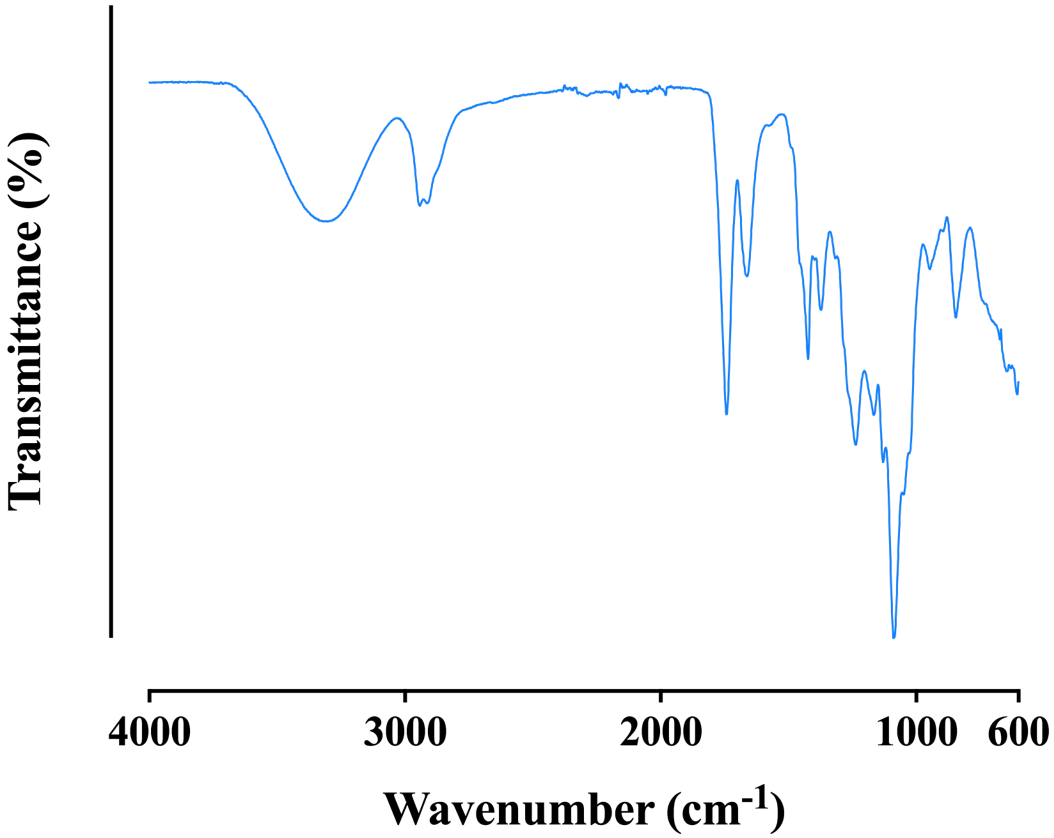

Figure S2: Representative ATR-FTIR spectrum of *C. glaziovii* freeze-dried extract.

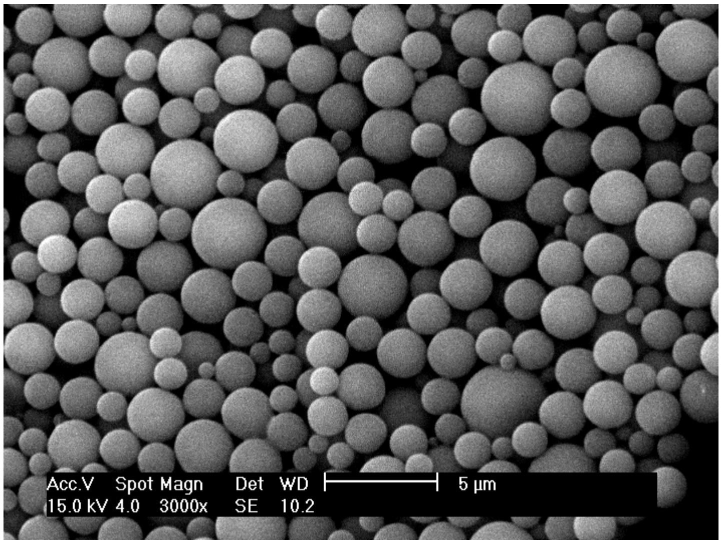

Figure S3: Representative scanning electron microscopy image of the *Cecropia glaziovii*-loaded PLGA microspheres (3000x).

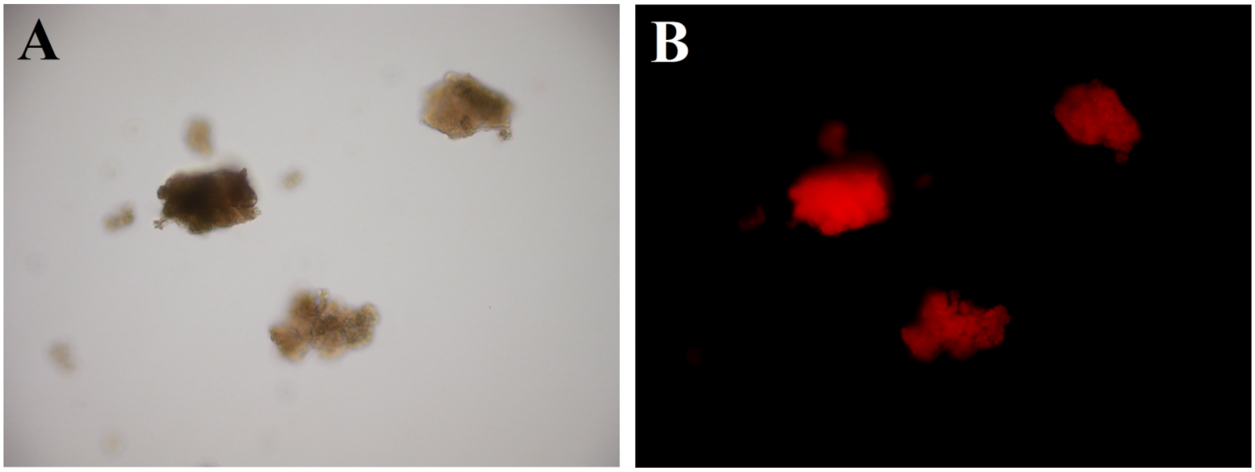

Figure S4: Representative (**A**) light microscopy and (**B**) epifluorescence microscopy images of *Cecropia glaziovii* freeze-dried extract particles (40x magnification).

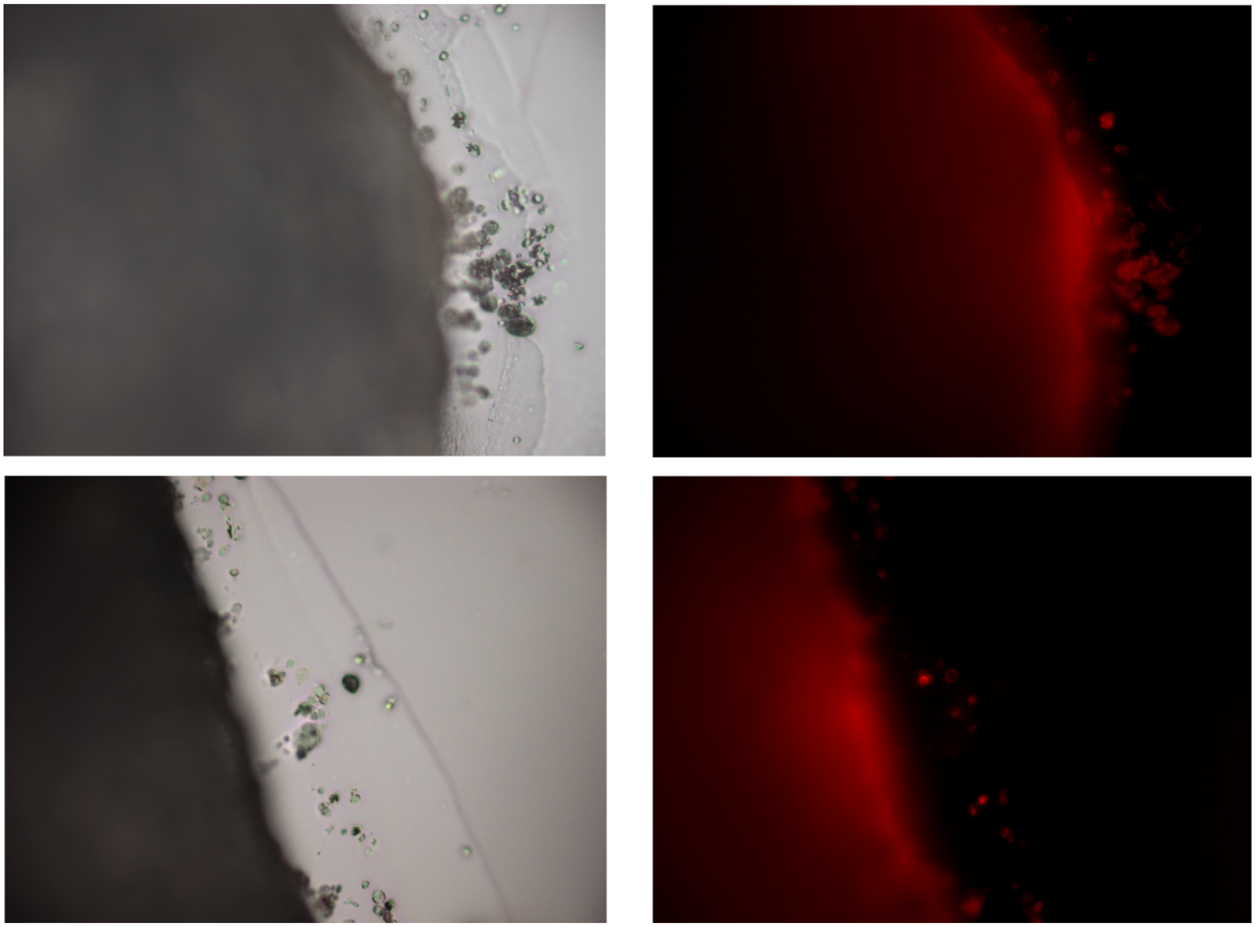

Figure S5: Representative images of cryosectioned microspheres-coated pellets under light (left column) and epifluorescence microscopy (right column).

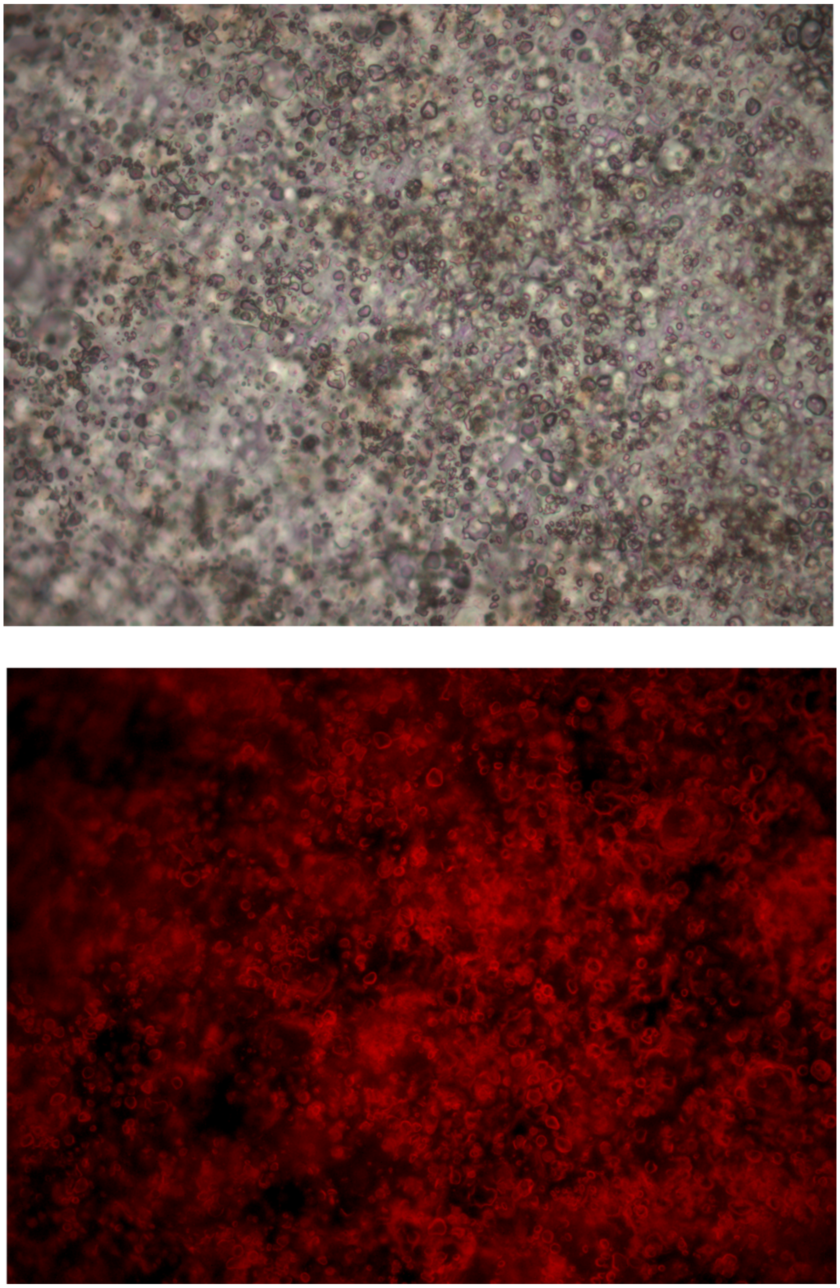

Figure S6: Light microscopy and epifluorescence microscopy of the coating film extracted from the microspheres-coated pellets using a scalpel.
